# Radial Endobronchial Ultrasound to Diagnose a Case of Non-Hodgkin’s Lymphoma in the Lung: A Case Report and Literature Review

**DOI:** 10.7759/cureus.55183

**Published:** 2024-02-28

**Authors:** Vineet Simhan, Srivatsa Lokeshwaran, Nitesh Gupta, Uzair Baig, Susmita Rakshit

**Affiliations:** 1 Pulmonary and Critical Care Medicine, Aster Hospital Whitefield, Bangalore, IND; 2 Interventional Pulmonology, Aster Hospital Whitefield, Bangalore, IND; 3 Pulmonary, Critical Care and Sleep Medicine, Vardhman Mahavir Medical College and Safdarjung Hospital, Delhi, IND; 4 Pulmonology, Aster Hospital Whitefield, Bangalore, IND; 5 Pathology, Aster Hospital Whitefield, Bangalore, IND

**Keywords:** non-hodgkin’s lymphoma, pulmonary lymphoma, lung malignancy, non-resolving pneumonia, radial endobronchial ultrasound (r-ebus)

## Abstract

Non-Hodgkin’s lymphomas (NHLs) are a heterogeneous group of lymphoproliferative malignancies that are very rarely seen in the lung. Although they generally have a favorable prognosis, the clinical symptoms and most efficient methods of diagnosis have not yet been clearly defined. This report highlights an interesting case wherein a 75-year-old male who presented with complaints of fever, cough, and generalized weakness for three weeks was diagnosed and treated as a case of pneumonia. He did not respond to conventional treatment with antibiotics and antipyretics. Hence, computed tomography of the thorax was done which showed consolidation in the right lower lobe along with a few enlarged right hilar nodes. To evaluate this unresolved pneumonia, he was further evaluated with a radial endobronchial ultrasound (EBUS) and biopsy, which helped in arriving at a diagnosis of NHL. This case illustrates the significance of advanced interventions such as radial EBUS to identify the exact etiology of the lesions. This is the first case to document the ultrasound images of NHL in the lung, obtained using a radial EBUS.

## Introduction

Most patients with non-Hodgkin’s lymphoma (NHL) of the lung are asymptomatic and are diagnosed following routine X-ray screenings, showing infiltrates in the lung. However, if symptoms are present, they are most commonly non-specific and include fever, weight loss, and night sweats. Respiratory symptoms, if present, include cough, pain or pressure sensation in the chest, or dyspnea [1]. Due to this non-specific presentation, arriving at a diagnosis of NHL in the lung may be missed in a large proportion of patients. X-rays generally show pulmonary abnormalities resembling pneumonia, such as consolidation with air bronchograms. As a result, patients with NHL who are treated as pneumonia see no improvement in their symptoms or X-rays [2].

A thorough evaluation of suspected cases should include computed tomography (CT) of the thorax to assess and identify potential hilar and mediastinal lymphadenopathy which may prompt further investigation to support the diagnosis of NHL. Radial-probe endobronchial ultrasound (EBUS) has emerged as a widely accepted procedure that can help localize and guide tissue sampling for an accurate diagnosis of peripheral pulmonary nodules [3,4]. Here, we discuss an interesting case of NHL in the lung which presented as a classic case of pneumonia. Due to the unresolved nature even after treatment with antibiotics, we took the patient up for radial EBUS with biopsy to arrive at an accurate diagnosis.

## Case presentation

**Figure 1:**
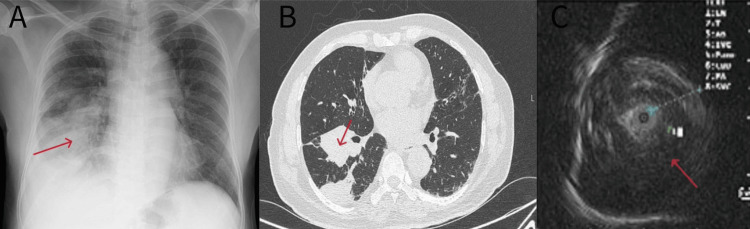
(A) Chest X-ray showing consolidation in the right lower lobe (arrow). (B) High-resolution CT of the thorax demonstrating evidence of subpleural and peribronchial consolidation in the right lower lobe (arrow). (C) R-EBUS image demonstrating central hypoechoic mass with linear arcs and dots within the lesion (arrow), surrounded by a hyperechoic margin, thus confirming R-EBUS within the lesion. CT: computed tomography; R-EBUS: radial endobronchial ultrasound

A 75-year-old man with no comorbidities was hospitalized with fever, cough, and loss of weight and appetite for three weeks. Physical examination was significant for a temperature of 100.2°F, normal vesicular breath sounds with bronchial breath sounds in the right infrascapular region, no superficial lymph nodes, and oxygen saturation of 95% in room air. Chest X-ray revealed consolidation in the right lower zone, as demonstrated in Figure 1, Panel A. A provisional diagnosis of right pneumonia was made. He was treated with a course of antibiotics (intravenous ceftriaxone and oral macrolides) for 10 days, despite which the symptoms persisted, leading to further investigations. The sputum examination was negative for acid-fast bacilli, and *Mycobacterium tuberculosis* was not detected in GeneXpert MTB/RIF assay. CT of the thorax revealed a consolidation in the right lower lobe along with a few areas in the right upper lobe (Figure 1, Panel B). Mild heterogenous enhancement of the consolidation was noted, and there was no evidence of necrosis. There were a few enlarged right hilar lymph nodes, with the largest measuring 11 mm in short axis.

After obtaining consent from the patient, we decided to proceed with a transbronchial lung biopsy to establish a diagnosis under the guidance of radial EBUS given unresolved pneumonia. CT planning was done using our InstaRISPACS software to identify the leading airway from the right main bronchus into the consolidated changes in the right lower lobe which was identified as the RB8b segment. Under general anesthesia and with the help of fluoroscopic guidance, Ultrasonic probe 1.4 mm UM-S20-17S (Olympus) was introduced into the proposed area (RB8b), as suggested by the CT planning through the working channel of the Olympus BF-MP-190F bronchoscope (3.7 mm OD, 1.7 mm ID). We identified hyperechoic dots and linear arc patterns with blood vessels (Type IIb) surrounding the probe in the right lower lobe (Figure 1, Panel C).

**Figure 2:**
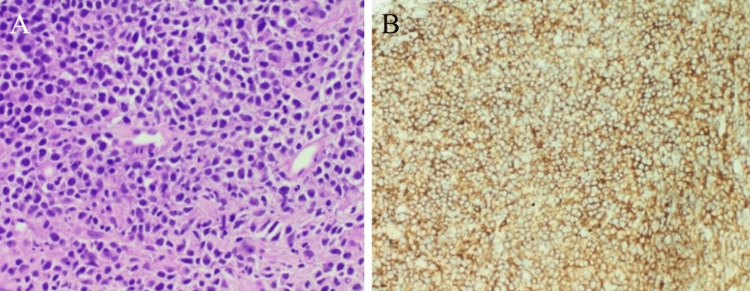
Endobronchial tissue fragments lined by ciliated columnar epithelium with underlying stroma consisting of monomorphic sheets of medium-to-large atypical lymphoid cells with round-to-oval vesicular nuclei with prominent nucleoli traversed by fibrovascular strands, atypical mitoses seen with no evidence necrosis. Staining agent used: hematoxylin and eosin (A). Atypical lymphoid cells which are diffusely positive for Immunohistochemistry marker CD20 (magnification ×40) (B).

The radial EBUS was removed, following which a 1.1 mm cryoprobe was introduced into the working channel. Using a freeze time of four to five seconds, we obtained a transbronchial cryolung biopsy from the area. The tissue obtained was sent for histopathological analysis. Bronchoscopy and bronchoalveolar lavage were obtained and sent for acid-fast stain and bacterial and fungal culture to determine etiology. Histopathology showed endobronchial tissue fragments lined by ciliated columnar epithelium, with monomorphic sheets of medium-to-large atypical lymphoid cells with round-to-oval vesicular nuclei with prominent nucleoli traversed by fibrovascular strands (Figures 2A, 2B).

Histology was suggestive of NHL and immunohistochemistry markers were sent for further subtyping which showed that leukocyte common antigen was diffusely positive, CD3 was reactive, and CD20 diffusely positive. This helped in confirming our diagnosis of NHL of B-cell origin. A positron emission tomography (PET) scan showed metabolically active lesions in the right upper and lower lobes of the lung, as well as involvement in the subcarinal, paraesophageal lymph nodes, and spleen, helping to confirm the staging as stage III lymphoma.

## Discussion

Primary NHL of the lung is an uncommon condition, accounting for only 0.4% of all lymphomas, in contrast with secondary lung involvement, which is more prevalent in individuals with a lymphoma history (incidence 25% to 40%) [1]. Two studies by Ferraro et al. and Li et al. showed that out of 92 NHL patients, 43.4% (40 patients) were asymptomatic and diagnosed only after routine radiography, while the remaining 56.5% (52 patients) had either systemic or respiratory symptoms, most commonly fever, weight loss, night sweats, dyspnea, and cough [1,2]. Our patient had unresolved cough, breathlessness, and fever for 22 days, despite treatment with antibiotics and other supportive medications, due to which we opted to biopsy the lesion using radial EBUS as a navigation tool. Radial-probe EBUS can precisely locate peripheral pulmonary nodules or masses and allows for clear visualization of bronchial and vascular structures, reducing the incidence of complications such as bleeding, while avoiding areas of necrosis to improve diagnostic yield [3,4]. Kurimoto et al. [5] classified EBUS patterns into three distinct categories with six subsets, as shown in Table 1.

**Table 1:** Classification and characteristics of the types of radial endobronchial ultrasound images.

Classification	Characteristics	Etiology
Type I	Homogenous pattern	
Ia	With patent vessels and bronchioles	Pneumonia
Ib	Without vessels and bronchioles	Tuberculoma/Organizing pneumonia
Type II	Hyperechoic sots and linear arc pattern	
IIa	Without preserved blood vessels	Well-differentiated adenocarcinoma
IIb	With preserved blood vessels	Well-differentiated adenocarcinoma
Type III	Heterogenous pattern	
IIIa	With hyperechoic dots and short lines	Moderately differentiated adenocarcinoma/squamous cell carcinoma
IIIb	Without hyperechoic dots and short lines	Poorly differentiated adenocarcinoma

Chao et al. suggested that four distinct sonographic features (lobulation, margins, blood vessels, and air bronchogram) can help determine whether a lesion is benign or malignant [6]. In our case, radial EBUS showed hyperechoic dots and a linear arc pattern with blood vessels (Type IIb).

Extranodal lymphomas in the lung are rare (usually low-grade B-cell types) and originate from mucosa-associated lymphoid tissue from the bronchus [7]. Histopathological analysis of the biopsied sample in our case showed an endobronchial lesion composed of monomorphic medium-to-large atypical lymphoid cells with oval-to-round nuclei with fine chromatin. CD20 was positive in the atypical lymphoid cells and CD3 showed a reactive pattern. Based on the above findings, a diagnosis of diffuse large B-cell lymphoma (DLBCL) type of NHL was given.

Treatment of NHL commonly involves a combination of chemotherapy, radiotherapy, and immunotherapy and is based on the stage and tumor burden. Monoclonal therapy and radioimmunoconjugate therapy have become the standard of care for the treatment of indolent lymphomas, while aggressive lymphomas are generally treated using either the CHOP (cyclophosphamide, doxorubicin, vincristine, and prednisone) or rituximab-CHOP regimen [8]. Rituximab (anti-CD20 agent)-based chemotherapy regimens have shown very favorable outcomes in patients with DLBCL [9,10]. Anthracyclines such as doxorubicin have been associated with increased incidence of dose-related cardiotoxicity due to myocarditis, due to which in our institute we initiate chemotherapy with the RCEOP (rituximab, cyclophosphamide, etoposide, vincristine, and oral prednisolone) regimen [11]. Our patient was successfully treated with a total of six cycles of the RCEOP regimen which yielded a favorable outcome with significant symptomatic improvement and repeat PET showed a reduction in metabolically active lesions.

## Conclusions

NHL of the lung is a very rare condition, often presenting with non-specific symptoms that can lead to delayed diagnosis and treatment. Our recent case underscores the crucial role of radial EBUS as a pivotal tool in obtaining a biopsy sample in a patient. Its ability to provide real-time imaging guidance during bronchoscopic procedures offers clinicians a minimally invasive and precise means to sample suspicious lesions within the lung parenchyma. By facilitating accurate tissue acquisition, EBUS enhances diagnostic yield and ultimately guides treatment strategies for patients. The utility of radial EBUS not only lies in its diagnostic efficacy but also in its potential to minimize procedural risks and expedite patient management, thus underscoring its indispensable role in the multidisciplinary approach to managing NHL of the lung.

## References

[1] Ferraro P, Trastek VF, Adlakha H, Deschamps C, Allen MS, Pairolero PC (2000). Primary non-Hodgkin's lymphoma of the lung. Ann Thorac Surg.

[2] Li G, Hansmann ML, Zwingers T, Lennert K (1990). Primary lymphomas of the lung: morphological, immunohistochemical and clinical features. Histopathology.

[3] Jacomelli M, Demarzo SE, Cardoso PF, Palomino AL, Figueiredo VR (2016). Radial-probe EBUS for the diagnosis of peripheral pulmonary lesions. J Bras Pneumol.

[4] Lee SC, Kim EY, Chang J, Lee SH, Han CH (2020). Diagnostic value of the combined use of radial probe endobronchial ultrasound and transbronchial biopsy in lung cancer. Thorac Cancer.

[5] Kurimoto N, Murayama M, Yoshioka S, Nishisaka T (2002). Analysis of the internal structure of peripheral pulmonary lesions using endobronchial ultrasonography. Chest.

[6] Chao TY, Lie CH, Chung YH, Wang JL, Wang YH, Lin MC (2006). Differentiating peripheral pulmonary lesions based on images of endobronchial ultrasonography. Chest.

[7] Shaikh AB, Waghmare S, Koshti-Khude S, Koshy AV (2016). Unusual presentation of non-Hodgkin's lymphoma: case report and review of literature. J Oral Maxillofac Pathol.

[8] Ansell SM, Armitage J (2005). Non-Hodgkin lymphoma: diagnosis and treatment. Mayo Clin Proc.

[9] Singh R, Shaik S, Negi BS, Rajguru JP, Patil PB, Parihar AS, Sharma U (2020). Non-Hodgkin's lymphoma: a review. J Family Med Prim Care.

[10] Cordier JF, Chailleux E, Lauque D (1993). Primary pulmonary lymphomas. A clinical study of 70 cases in nonimmunocompromised patients. Chest.

[11] Al-Sarayfi D, Meeuwes FO, Durmaz M (2022). R-CEOP as first-line treatment for anthracycline-ineligible patients with diffuse large B-cell lymphoma. Blood Cancer J.

